# Lowering Activation Barriers to Success in Physical Chemistry (LABSIP): A Community Project

**DOI:** 10.1021/acs.jpca.3c07015

**Published:** 2023-12-19

**Authors:** Carlos R. Baiz, Robert F. Berger, Kelling J. Donald, Julio C. de Paula, Stephen D. Fried, Brenda Rubenstein, Grace Y. Stokes, Kana Takematsu, Casey Londergan

**Affiliations:** † Department of Chemistry, University of Texas at Austin, Austin, Texas 78712, United States; ‡ Department of Chemistry, Western Washington University, Bellingham, Washington 98225, United States; § Department of Chemistry, University of Richmond, Richmond, Virginia 23173, United States; ∥ Department of Chemistry, Lewis & Clark College, Portland, Oregon 97219, United States; ⊥ Department of Chemistry, Johns Hopkins University, Baltimore, Maryland 21218, United States; # T. C. Jenkins Department of Biophysics, Johns Hopkins University, Baltimore, Maryland 21218, United States; 7 Departments of Chemistry and Physics, Brown University, Providence, Rhode Island 02912, United States; 8 Department of Chemistry & Biochemistry, Santa Clara University, Santa Clara, California 95053, United States; 9 Department of Chemistry, Bowdoin College, Brunswick, Maine 04011, United States; 10 Department of Chemistry, Haverford College, Haverford, Pennsylvania 19041, United States

## 1 MOTIVATION

Physical chemistry is a major pillar of the undergraduate curriculum. In many four-year colleges and universities in the United States, the chemistry major requires two semesters of physical chemistry (and their associated laboratory courses), during which students are often first exposed to the foundational ideas and equations of quantum mechanics, thermodynamics, and kinetics.^1,2^ Two semesters of physical chemistry are standard requirements in many departments for undergraduate majors. Per ACS Guidelines, however, only one semester of Physical Chemistry is required as part of the coursework for ACS Approved Bachelor’s degrees.^3^

**Figure 1:**
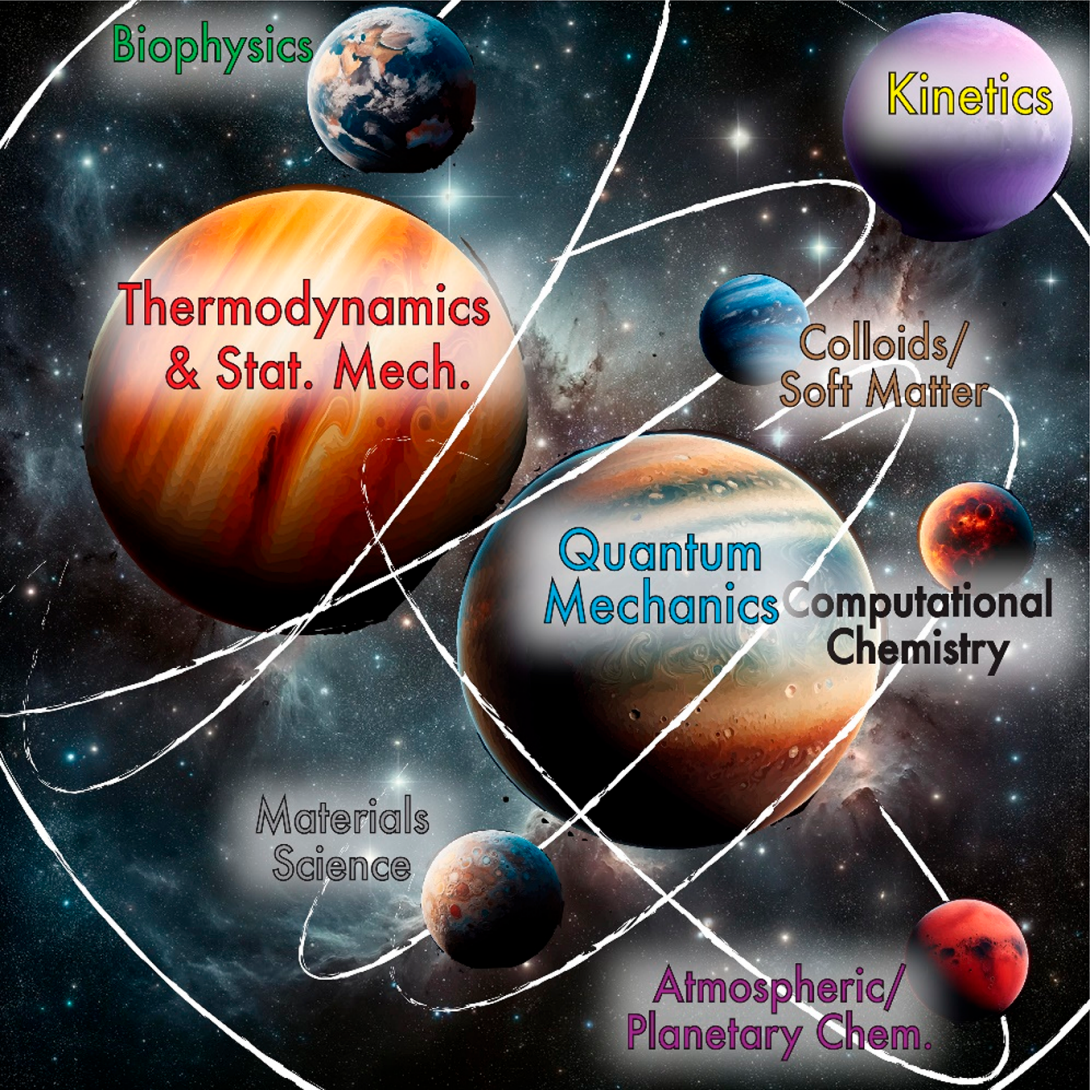
A “planetary model” of physical chemistry topics, in which two central concepts (thermodynamics/statistical mechanics and quantum mechanics, the two “gas giants”) provide the foundation for a broad variety of topics and areas of current research (smaller rocky planets, ocean worlds, and moons). Community consensus in our workshops points to the central importance of the “two gas giants” model but also can provide a great degree of instructor and student freedom in exploring the remainder of the space in our subdiscipline.

Over the past few decades, physical chemistry as a research discipline has grown significantly. Compared to their original focus on the structure and reactivity of small molecules in the gas phase, physical chemists today now make pivotal contributions to fields as diverse as biophysics, soft matter physics, materials science and engineering, environmental science, atmospheric and planetary science, and catalysis and surface science, to name a few (Figure 1). While these specialized subfields still draw (as they have for over a century)^4^ from the two core curricular disciplines of thermodynamics and quantum mechanics, physical chemistry instruction has not kept pace with this emerging diversity and expansion of the field. Typical course syllabi and popular textbooks remain focused on the topics and examples that defined physical chemistry in the 19th and early 20th centuries. In addition, physical chemistry syllabi tend to be content-heavy and textbooks encyclopedic, which can be problematic when adapting the course to distinct formats as required by institutional or curricular needs (e.g., semester or quarter systems, courses for majors or prehealth students). Moreover, teaching resources of this type can reinforce traditional (and not always empowering) pedagogy and create barriers toward adopting newer evidenced-based teaching practices that lead to improved learning outcomes, many of which have been known for years but have not been widely adopted.^5−10^

It is probably not controversial to argue that this status quo should not continue indefinitely. On one hand, instructors of physical chemistry increasingly come from a broad range of specialties and may identify primarily in their research with allied subjects (e.g., as biophysicists, materials scientists, etc.) rather than as physical chemists. This ought to be seen as an asset rather than a liability, as these instructors can enrich physical chemistry courses by drawing examples and applications from across the contemporary research literature. In parallel, undergraduates seeking degrees in chemistry form an increasingly diverse cohort, with a broader range of backgrounds, interests, and career goals. In addition to its primary purpose of training future chemists, the chemistry curriculum provides excellent foundational training in medicine, sustainability, numerical and statistical analysis, and technology. While these specialties may connect to physical chemistry to varying degrees, physical chemistry’s status as a required component of the major imbues it with the responsibility to provide meaningful training to students with diverse academic interests. Moreover, given its earned reputation as one of the most difficult subjects in the chemistry major, physical chemistry can also act, unfortunately, as a gatekeeper, if not a deterrent, to completing a degree in chemistry. Its unintentional status as a common attrition point in the chemistry training pipeline for students who are otherwise passionate about chemistry should give physical chemistry instructors pause. If teaching practices are not dynamic and inclusive, they will likely impact negatively the diversity of students who obtain chemistry degrees and go forward successfully in the chemical sciences.

All of these factors motivated a number of us (including the authors) to convene a group of physical chemistry instructors to form LABSIP, or *Lowering Activation Barriers to Success in PChem*. The overarching goal of the LABSIP Collaborative is to promote systemic change that will enable more students and instructors to have successful experiences learning and teaching physical chemistry. We aim to achieve this goal by generating public resources and creating a vibrant and diverse community of practice. As described in the following, we have found substantial interest within the physical chemistry instructor community to propel this project forward by addressing a common set of challenges. We next will describe the activities that LABSIP has initiated during its first year, and then report what we have learned from these initiatives regarding an emerging community-wide consensus on challenges. We will also report innovative strategies and resources that can address those common challenges. In addition to serving as LABSIP’s first-year activity report and description of its future goals, this Viewpoint doubles as an open invitation to all physical chemistry instructors to become members of and contributors to LABSIP.

## 2 INTRODUCTION TO THE LABSIP COLLABORATIVE

The Research Corporation for Science Advancement (RCSA) supports early career faculty in chemistry, physics, and astronomy with the Cottrell Scholar Award and brings them together annually in Tucson, Arizona, to brainstorm about improving teaching, research, and mentoring in the sciences. Among the many physical chemists at the July 2022 meeting, which was the first in-person meeting post-COVID, the need to think more deeply about how and what we teach in physical chemistry courses became a vibrant topic of discussion; the LABSIP Collaborative grew out of those discussions. The group of 12 faculty members involved in the collaborative shared an interest in building a community of physical chemists, instructors who value excellence and inclusivity in chemical instruction and who wished to think more deeply, along with colleagues across the country, about pedagogical frameworks that enrich students’ appreciation and understanding of modern physical chemistry and its relationships to other fields. The initial group that obtained funding for LABSIP from RCSA shortly after the 2022 Cottrell Scholar meeting represented a wide range of institutions (liberal arts colleges, regional comprehensive universities, and research universities), career stages (assistant, associate, and full professors), research foci (spectroscopy, biophysics, and computation), and physical chemistry course schedules and formats (semesters and quarters, courses for chemistry majors and prehealth students).

Since its establishment, the LABSIP Collaborative has held three workshops: two online and one in-person. At the 2 hr online workshop held in November 2022, approximately 170 attendees—faculty members teaching physical chemistry at a wide range of colleges from across the United States—discussed challenges, priorities, and (through a series of short “lightning” talks) innovative ideas arising from their teaching of physical chemistry. That event was followed by a 3 hr online workshop in June 2023 in which a substantial amount of community feedback was collected and prioritized to determine how LABSIP could provide the most benefit to the community. Recordings of key parts of these workshops are available on the LABSIP YouTube channel (link via http://labsip.org). Discussions at the two online meetings showed that, surprisingly, faculty teaching physical chemistry at very different institutions have similar objectives, face similar challenges, and are committed to improving the effectiveness of their physical chemistry teaching in both the mode of instruction and the balance of content. At a two-day in-person workshop in July 2023, again in Tucson, Arizona—working against record high temperatures—a smaller group of participants that included but was not limited to members of the core collaborative (see Table S1 for a full list of participants) began organizing and planning initial actions and resources, many of which will be discussed below.

## 3 WHAT WE HAVE LEARNED SO FAR

A striking theme emerged from our November 2022 and June 2023 workshops and many recent conversations with the wider community: *There is widespread agreement regarding the challenges that face physical chemistry instructors and the need to establish physical chemistry communities of practice.* To better understand community needs and challenges, during the first LABSIP workshop held online in November 2022, we asked participants the following three questions:

- What are the challenges that instructors and students face with student learning and successful completion of physical chemistry courses?
- What resources would be most useful to help overcome these challenges?
- What specific content is most important for your physical chemistry courses?

The discussion of these prompts and the subsequent workshops that they inspired have pointed to an emerging consensus on the following key topics in the community:

### 3.1 A Need (and Desire) for a Vibrant Community of Practice

In the first workshop, the importance and desire for a community of physical chemistry instructors became evident quickly. Many participants were excited about the increasing scientific and student diversity in the broad field of physical chemistry, but they were unsure about how best to approach or address changes in the curriculum. While many expressed a desire to modernize or alter their courses, they also acknowledged the challenge and tacit expectation of covering a large amount of content in their courses, purchasing and getting trained on modernized lab equipment, and finding the time and energy to develop new materials. These hurdles were only worsened for instructors whose home institutions were facing budget cuts or falling student enrollments. Participants also strongly noted the challenges of teaching students with different mathematical and/or computational skills. While the importance of these skills was recognized by their departments, participants often felt that, as physical chemists, they were addressing these challenges alone.

In the discussions that followed, concrete solutions were not proposed; instead, participants began to share what resources or support could make these challenges easier to overcome. Junior faculty expressed a desire for more teaching mentorship and a shared repository of resources, while senior faculty added the need for professional development workshops focused on modernizing the physical chemistry curriculum. Several participants highlighted the work and progress made by current microgroups in physical chemistry (e.g., POGIL–PCL,^9,11,12^ PIPER,^13^ the ESCIP project,^14−16^ the MERCURY^17^ consortium, and MolSSI Education^18,19^), yet it became clear that not everyone was aware of these resources, and all agreed that it would be helpful to create a centralized location to connect groups with each other and to the broader community of physical chemistry instructors.

A clear consensus of the November 2022 workshop was how helpful and important it was for members of the physical chemistry community to talk and connect frequently with each other about the curriculum. Beyond conversations about frustrations and challenges, there were also exchanges of ideas (and much-needed laughter and support). Instructors at all levels were eager to learn about not only new teaching strategies and material for their classrooms but also about strategies to advocate more effectively for changes in the curriculum and policies in their home department and at the regional and national levels. For members who often felt isolated or siloed in their home departments, the main highlight of the workshops was simply having a chance to talk to another person in a meaningful manner about the physical chemistry curriculum and its future. Like our students, we feel a real need and desire to connect to a larger community.

With the need for a greater shared community enunciated in all of our events to date, we have generally been struck by the level of consensus among physical chemistry instructors across a broad array of institutions. We did not anticipate the high levels of both solidarity and shared opinions across our community. Points of consensus have included a clear and finite set of shared challenges and some strongly shared opinions about the content and competencies that could be the focus of re-envisioned physical chemistry courses.

### 3.2 Consensus on “Essential” Course Content

**Figure 2:**
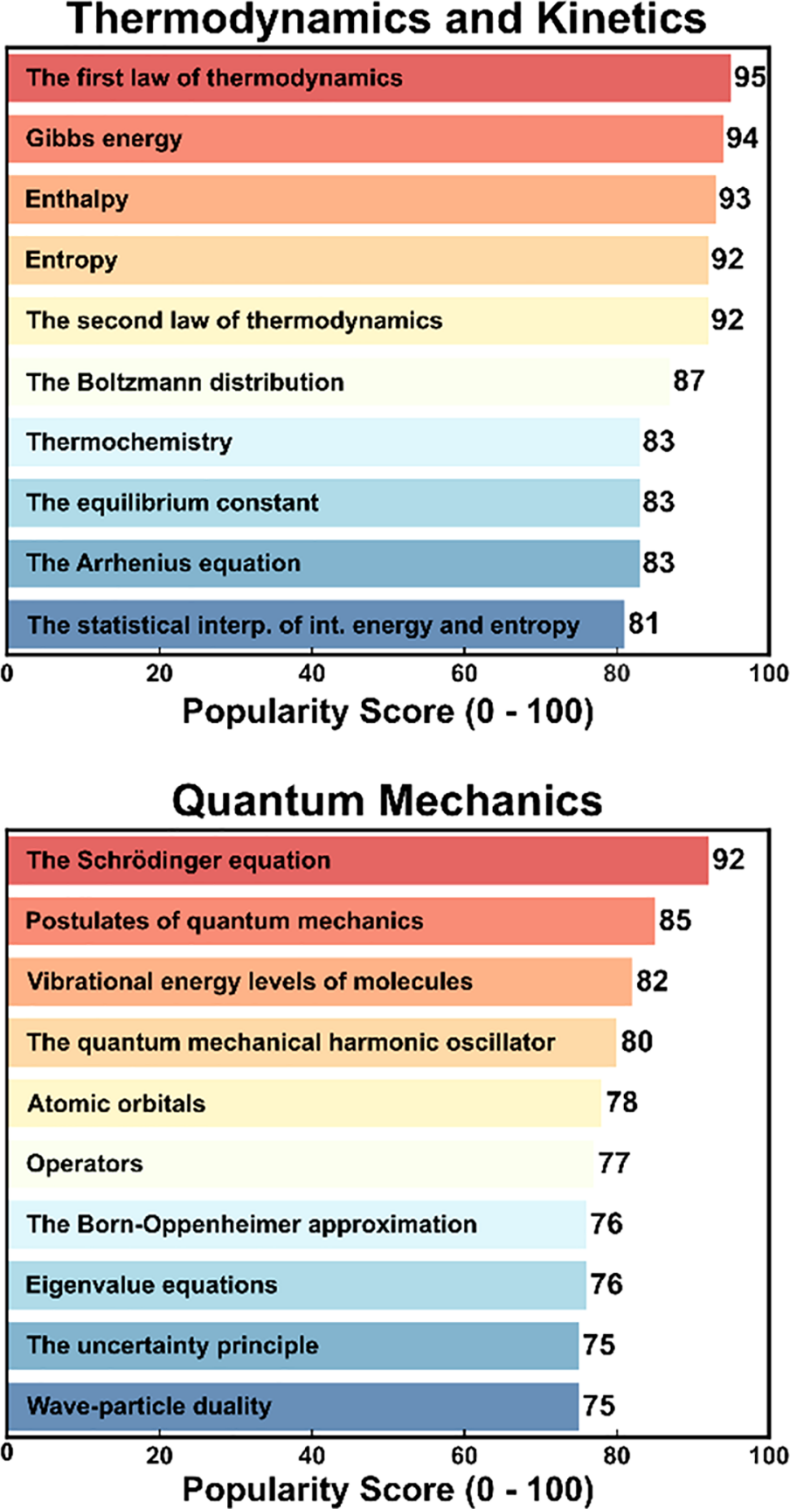
Ten most important topics in Thermodynamics and Kinetics (upper) Quantum Mechanics (lower) that the community identified for a “core” physical chemistry curriculum.

At the November 2022 meeting, after discussion of the aforementioned prompts, we conducted two real-time polls (one on thermodynamics topics and one on quantum chemistry topics) asking the ∼170 online participants which they would prioritize in their ideal physical chemistry curriculum. To do so, we employed the AllOurIdeas online platform^20^ (Figure 2), which enables users to compare two topics and upvote one over the other using the topic headings from the most recent edition of *Atkins’ Physical Chemistry* by Atkins and de Paula.^21^ In particular, we asked participants two questions: “*What thermodynamics, statistical mechanics, kinetics, and materials topics are most important in physical chemistry?*” and “*What quantum chemistry topics are most important in physical chemistry?*” The results emerged nearly immediately: the community valued “core” ideas and concepts over more applied topics.

As depicted in Figure 2, participants viewed such foundational concepts as the First Law of Thermodynamics, Gibbs free energy, enthalpy, entropy, the Second Law of Thermodynamics, the Boltzmann distribution, and the Arrhenius equation as the most important topics in classical physical chemistry, including a range that covers thermodynamics, statistical mechanics, and chemical kinetics. In contrast, more specialized topics such as Tafel plots, the Butler–Volmer equation, surface films, and the magnetic properties of solids were listed as much less important. Similarly, most participants viewed the Schrödinger equation, postulates of quantum mechanics, vibrational energy levels, the quantum mechanical harmonic oscillator, and eigenvalues as being the most important topics in quantum chemistry, while Doppler broadening, NMR and solid-state NMR, and EPR were deemed much less important.

In a subdiscipline with essentially two central content ideas upon which everything is built (perhaps two and a half with the inclusion of kinetics, as seen in Figure 1), such clear community consensus is heartening because it provides a potential shared path to reimagining physical chemistry courses. A curriculum that is more focused will no longer feel to students like a march through an endless series of equations and textbook chapters, but instead intentionally emphasize core ideas and then use the remaining space and time to engage students in applied topics of the greatest interest to them and their instructors. Tables S2 and S3 show scores for all of the topics identified by the two AllOurIdeas polls.

Based on these findings, our in-person workshop in July 2023 (see Table S1 for participant roster) worked to suggest minimal-content cores that could be used for physical chemistry courses of varying formulations, including single-term thermodynamics and quantum mechanics courses and single-semester comprehensive “introductory physical chemistry” courses. The goal of developing these cores was not to dictate which topics to cover (and not to cover) to instructors in their courses but rather to offer outlined examples of course plans that could provide instructors and students with greater space for originality, agency, and current relevance.

These content cores, which our in-person group of representative physical chemistry instructors designed, quickly suggest that physical chemistry courses need not be as voluminous and intimidating to students as they often are. The “shared core” ideas, which can then be surrounded by more applied, current research, or news-oriented topics, also point to a clear path forward for textbooks in physical chemistry (or the open educational resources that might replace them) in the medium and long-term. “Skinny” core-based texts or textbook-like resources could be complemented by applied topic-oriented modules, giving instructors and students with different goals and backgrounds the freedom and initiative to choose their paths forward. The result would be a more efficient way to learn how to *do* physical chemistry by illustrating and enacting *what physical chemists actually do* in their research and their engagement with the world around them.

### 3.3 Content-Independent Learning Goals

**Chart 1:**
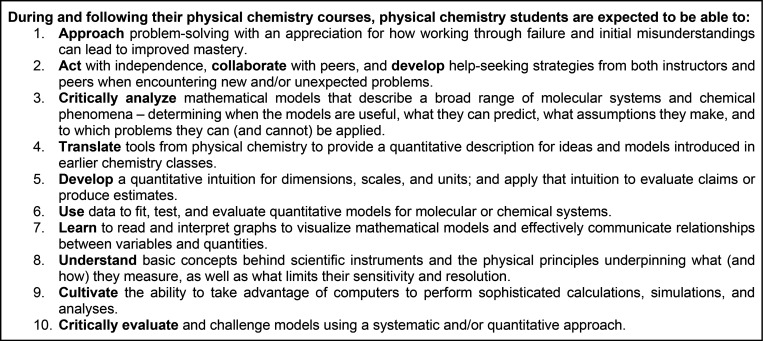
Ten Content-Independent Learning Goals for Physical Chemistry

The principle of “inverted course design” advises instructors to design their syllabi as follows:^22,23^ (a) identify what you want students to be able to ***do*** after successfully completing the course; (b) identify what forms of assessment will enable you to evaluate whether students have mastered those competencies; and (c) identify which lessons or exercises will enable students to perform well on those assessments. This philosophy is termed “inverted” or “backward” to contrast it with the seemingly more obvious approach of beginning course design by filling a syllabus with content. Because physical chemistry is often experienced as a content-heavy course with comprehensive textbooks, it can be particularly challenging for instructors to engage with higher-level learning objectives in the course. When confronted with the question, “*What do I want my students to be able to do after successfully completing physical chemistry?*,” the immediate answers that jump to mind for many are topical, such as “Students should be able to solve Schrödinger’s equation” or “Students should be able to calculate entropy changes.” While these are not inconsequential goals, the LABSIP Collective reflected at its in-person workshop in Tucson in July 2023 on some of the higher-level learning goals that can be accomplished by teaching physical chemistry courses. These ten so-called content-independent learning goals (CILGs) are enumerated in Chart 1. Importantly, it was felt that these learning goals were invariant to course length (semester or trimester), student constituency (e.g., chemistry majors or prehealth majors), or any particular specialization. We offer these goals formulated in ways that might inspire assessment strategies beyond strongly content-bound exams and other traditional assessment rubrics.

LABSIP has published these content-independent learning goals on its website (http://labsip.org/), and a number of us have included this language in our syllabi to communicate to students our vision as instructors. The ten CILGs ultimately reflect that we suggest that there are things that physical chemists should be able to ***do*** and that these categories transcend emphasizing what physical chemists should be expected to ***know***. In the following, we offer some insight into the discussion that led to the list compiled in Chart 1.

The first two CILGs are meta-cognitive, meaning they are not specific to physical chemistry *per se*. At the same time, the group agreed that these skills are fundamental to success in physical chemistry. Because it can be particularly challenging, the first “real” physical chemistry course that a student encounters often represents a turning point at which many students who are not used to asking for help or working with peers will be “required” to do so to succeed. Instructors should embrace this and be transparent. For students, there’s nothing more demoralizing than finding something hard when an instructor says it should be easy. To develop a sense of belonging in the classroom, physical chemistry instructors should normalize the feelings that are invariably associated with struggling to grasp difficult course material and encourage students to see the experience as an opportunity to grow as learners and thinkers in ways they perhaps had not in previous courses.

The mathematical nature of aspects of physical chemistry is well-known and has to be considered in pedagogical innovations for the subdiscipline such as remote (synchronous and asynchronous) instruction or course-based undergraduate research experiences.^24−27^ Many of the CILGs are motivated by the fact that physical chemistry courses are the most mathematical in the chemistry major, which gives them the clear responsibility to hone chemistry students’ quantitative reasoning skills. A theme that frequently arose in our discussions is the importance of imparting to students that mathematical models can make powerful predictions but can also be stringently tested. This ethos is imbued in CILGs 3, 5, 7, and 10.

Discussions on the role and importance of computers, programming, and coding courted the most controversy among the working group tasked with finalizing the list of CILGs. Several physical chemistry instructors have expressed the view that physical chemistry should also be a platform for exposing chemistry students to basic computer programming, not only to introduce tools that are particularly germane to the modern practice of physical chemistry (e.g., electronic structure calculations, classical simulations of fluids and polymers, and data visualization and analysis) but also to teach broadly useful skills for future careers in STEM fields. Ultimately, it was felt that it was inappropriate to make such prescriptive recommendations for the reasons that students’ backgrounds and instructors’ know-how vary too much for such recommendations to be adopted widely. It is worth noting that a large and growing number of physical chemistry instructors *do* subscribe to the thinking that exposure to programming enriches physical chemistry education. To assist instructors who want to incorporate computing into syllabi, LABSIP intends to publish computational modules in online repositories and provide training resources to instructors less fluent in computer code (*vide infra*, section 4), following and promulgating the examples of other communities already present in this space such as ESCIP (Enhancing Science Courses by Integrating Python).^14^ Nevertheless, CILGs 6 and 9 reflect critical takeaways from a modern physical chemistry course. Instructors should introduce students to the important relationship between quantitative data and mathematical models (CILG 6): mathematical models can be compared to experimental data to test the model, further understand it, and even refute the model. These ideas can be introduced using basic tools (e.g., spreadsheets) and in a wide range of contexts (e.g., obtaining Δ*H* from the temperature dependence of equilibrium constants, estimating force constants from vibrational spectra, etc.).

The ninth learning goal asks instructors to show students that many important problems in physical chemistry (e.g., simulating a liquid) are sufficiently complex that they are much better suited to computer-based approaches than through derivations or calculations by hand. Another topic of discussion along these lines was the relative importance (or, for some, irrelevance) of by-hand calculus, especially in the contexts of core ideas in thermodynamics and quantum chemistry. While currently adopting several different approaches to the use of calculus in their courses, workshop participants agreed that we are teaching at a moment at which many of the CILGs can be achieved through multiple approaches, ranging from by-hand approaches to more software- or programming-based modalities. LABSIP is committed to providing a community space where innovative approaches using any quantitative modality can be showcased and shared.

As a final point, we emphasize that the content-independent learning goals of physical chemistry should be compiled into a living document: an offering to the community that inspires new approaches. These goals were assembled by a working group consisting of early LABSIP members, but we hope (and expect) LABSIP to expand; as it does, the content-independent learning goals ought to be revisited and revised. We, therefore, invite physical chemistry instructors with suggestions for changes based on their own teaching experience to communicate accordingly.

## 4 ONGOING EFFORTS AND FUTURE GOALS

As LABSIP is an emerging community, we encourage all physical chemistry instructors to join us (see Section 5 below). All members can benefit from the collective voice and efforts of the community. The need and desire to create a vibrant community is clear and instructors across multiple institutions have already joined. We look forward to continuing our growth and coalescence as our community matures.

The initial workshops brought together a diverse group of instructors, across a range of institutions. During these initial phases, we identified a common set of priorities for the community, and we began to build infrastructure and organize members around the following specific goals:

1. The most important goal that emerged from the community workshops is the need to “create a community” and enable members to connect. Toward this goal, we created a Discord server where members can freely discuss topics, share tips, post resources, and organize around specific ideas. The Discord server is the main channel of personal communication across and between LABSIP members.
2. In addition, in Fall 2023, we began piloting cohort-building centered around “communities of practice” with specific foci. These, for example, included a community composed of new and experienced instructors dedicated to coaching new faculty on how to survive their first year teaching physical chemistry.
3. Another high-priority goal identified by the participants was to begin assembling a set of physical chemistry teaching resources. To organize the resources, we sought to identify specific content-independent learning goals that would complement the “skinny core” curricula for thermodynamics and quantum mechanics described above. These materials provide a roadmap for focusing our future efforts toward developing and sharing resources with the community. We envision creating a physical chemistry teaching resource repository analogous to the resources available in other communities such as the VIPEr inorganic chemistry repository,^28−30^ POGIL instruction materials,^11,12,31^ or PIPER resources.^13^ This repository will contain not only pedagogical resources but also serve as an outlet to share tips, strategies, experiences, or lessons learned.
4. We began hosting in-person LABSIP meetups at the ACS National Meetings. Our first two meetups took place at the ACS Spring Meeting in Indianapolis (March 2023) and the ACS Fall Meeting in San Francisco (August 2023). Our next meetup will take place at the ACS Spring Meeting in New Orleans (March 2024) in concert with the “Innovative Teaching in Physical Chemistry” symposium in the PHYS division. These are informal events at which LABSIP members can meet one another, discuss needs and priorities, and share knowledge.

We hope that pursuing these goals collectively, as a community, will transform and grow the field by leading to physical chemistry courses that energize and inspire both students and faculty for decades to come.

## 5 HOW TO JOIN LABSIP

Those interested in joining the LABSIP community are welcome to subscribe to the email list and join the Discord server (instructions on our Web site: http://labsip.org). The email list is used to distribute community-wide announcements to all members at a typical frequency of approximately two emails per semester. Recent emails have included announcements of online workshops and meetups at ACS meetings and other events of interest to the community of physical chemistry instructors, sharing-out of common priorities, and invitations to join our Discord server where more informal discussions take place.

As we seek to expand participation in the LABSIP Collaborative via in-person and virtual meetings and on social media, it is of the utmost importance to welcome a diverse range of viewpoints and better represent the full range of institutions contributing to the discussion. To ensure that our future endeavors reflect the full breadth of the physical chemistry experience, LABSIP must include, for example, historically Black colleges and universities, Hispanic-serving institutions, Tribal colleges and universities, and all Carnegie classifications of institutions that offer physical chemistry. While the institutions represented in the LABSIP Collaborative are, to date, primarily in the United States, participation may expand internationally, as well, because physical chemistry is a discipline without borders. Even as educational approaches and formats may differ from one country to another, the engagement across boundaries will be of mutual benefit and may move us closer to our common goal of promoting inclusive excellence in modern physical chemistry instruction.

## Supporting Information Available

Participant list for LABSIP workshop, ranked lists of physical chemistry topics for AllOurIdeas polls (PDF)
