# Supplementary material for: Lowering Activation Barriers to Success in Physical Chemistry (LABSIP): A Community Project
Source: J Phys Chem A. 2023 Dec 19;128(1):3–9. doi: 10.1021/acs.jpca.3c07015 (PMC10788899; doi:10.1021/acs.jpca.3c07015)
Supplement: Supplementary file 1 — jp3c07015_si_001.pdf [file jp3c07015_si_001.pdf]

SUPPORTING INFORMATION

Lowering Activation Barriers to Success in Physical Chemistry (LABSIP): A Community Project

Carlos R. Baiz,[1] Robert F. Berger,[2] Kelling J. Donald,[3] Julio C. de Paula,[4] Stephen D. Fried,[5,6] Brenda Rubenstein,[7] Grace Y. Stokes,[8] Kana Takematsu,[9] Casey Londergan[10]*

1. Department of Chemistry, University of Texas at Austin, Austin, TX 78712, USA
2. Department of Chemistry, Western Washington University, Bellingham, WA 98225, USA
3. Department of Chemistry, University of Richmond, Richmond, VA 23173, USA
4. Department of Chemistry, Lewis & Clark College, Portland, OR 97219, USA
5. Department of Chemistry, Johns Hopkins University, Baltimore, MD 21218, USA
6. T. C. Jenkins Department of Biophysics, Johns Hopkins University, Baltimore, MD 21218, USA
7. Departments of Chemistry and Physics, Brown University, Providence, RI 02912, USA
8. Department of Chemistry & Biochemistry, Santa Clara University, Santa Clara, CA 95053, USA
9. Department of Chemistry, Bowdoin College, Brunswick, ME 04011, USA
10. Department of Chemistry, Haverford College, Haverford, PA 19041, USA

*corresponding author: clonderg@haverford.edu

Table S1. Participant list for in-person LABSIP workshop at the Westin La Paloma in Tucson, AZ on July 18-19, 2023.

Name	Institution
Carlos Baiz	University of Texas at Austin
Leah Bendavid	Vassar College
Robert Berger	Western Washington University
Connor Bischak	University of Utah
Kristi Closser	California State Fresno
Linda Columbus	University of Virginia
Jahan Dawlaty	University of Southern California
Julio de Paula	Lewis & Clark College
Kelling Donald	University of Richmond
Stephen Fried	Johns Hopkins University
Theodore Goodson	University of Michigan
Heidi Hendrickson	Lafayette College
Matthias Heyden	Arizona State University
Daniela Kohen	Carleton College
Jeremy Kua	University of San Diego
Casey Londergan	Haverford College
Todd Martinez	Stanford University
Brenda Rubenstein	Brown University
Juliane Simmchen	TU Dresden, Germany
Steve Singleton	Coe College
Grace Stokes	Santa Clara University
Kana Takematsu	Bowdoin College
Mary Van Vleet	Spelman College

Table S2. Ranked list of Physical Chemistry "Topics in Thermodynamics and Kinetics" from AllOurIdeas poll initiated during the LABSIP Workshop, November 2022. The topics are ranked in descending order of importance.

**Topic**	**Score (0 -100)**
The first law of thermodynamics	95
Gibbs energy	94
Enthalpy	93
Entropy	92
The second law of thermodynamics	92
The Boltzmann distribution	87
Thermochemistry	83
The equilibrium constant	83
The Arrhenius equation	83
The statistical interpretation of the internal energy and entropy	81
Integrated rate laws	79
The kinetic model of gases	78
Real gases	77
The third law of thermodynamics	76
The perfect gas	75
Phase diagrams of pure substances	73
Rate laws and rate constants	73
The steady-state approximation	73
Molecular energies from the Boltzmann distribution	71
The rate-determining step	69
Elementary reactions	68
The thermodynamics of mixing	68
Calorimetry	67
Molecular interactions	67
Maxwell relations	67
Reaction order	65
Helmholtz energy	65
The van 't Hoff equation	65
Adiabatic processes in perfect gases	64
Transition state theory (Eyring equation)	64
The relation between K and the partition function	63
Kinetic and thermodynamic control of reactions	63
Unimolecular reactions	60
Partial molar quantities	60
Ideal solutions	59
Collision theory	59
Consecutive elementary reactions	58

Molecular interactions in liquids	57
The Carnot cycle	57
Partition functions	56
The Gibbs-Duhem equation	54
The canonical ensemble	53
Activities	53
Phase diagrams of binary systems	52
The Nernst equation	52
Colligative properties	52
Ideal-dilute solutions	50
The phase rule	49
Mechanisms of heterogeneous catalysis	49
Photochemical processes	48
The Debye-Hückel limiting law	47
Pre-equilibria	45
Adsorption isotherms	45
Diffusion in liquids	45
Enzyme-catalysed reactions	44
Diffusion-controlled reactions	44
Electrochemical cells	41
Galvanic cells	41
Transport properties of a perfect gas	40
Mean activity coefficients	40
State-to-state reaction dynamics	39
The kinetic isotope effect	38
Distillation	37
Electrode potentials	37
Polymerization kinetics	37
Surface tension	36
Critical solution temperatures	34
Macromolecules	34
Phase diagrams of ternary systems	32
Resonance energy transfer	32
Covalent and molecular solids	31
The electrical properties of solids	30
Electric properties of molecules	30
Capillary action	28
The electrochemical series	28
Electron transfer in homogeneous systems (Marcus theory)	28
Molecular beams	28
Voltammetry	28
Electrolysis	27
Excess functions and regular solutions	27
Micelles and biological membranes	27

Metals	25
Eutectics	24
Colloids	23
Ionic solids	22
Quantum mechanical scattering theory	22
Dynamics processes at electrodes	22
X-ray crystallography	22
The RRK model	21
Crystal structure	21
Surface growth	20
The optical properties of solids	20
The mechanical properties of solids	18
The magnetic properties of solids	15
Neutron and electron diffraction	15
Surface films	15
The Butler-Volmer equation	14
Tafel plots	9

Table S3. Ranked list of Physical Chemistry “Topics in Quantum Mechanics” from AllOurIdeas poll initiated during the LABSIP Workshop, November 2022. The topics are ranked in descending order of importance.

**Topic**	**Score (0 - 100)**
The Schrödinger equation	92
Postulates of quantum mechanics	85
Vibrational energy levels of molecules	82
The quantum mechanical harmonic oscillator	80
Atomic orbitals	78
Operators	77
The Born-Oppenheimer approximation	76
Eigenvalue equations	76
The uncertainty principle	75
Wave-particle duality	75
Expectation values	74
Spin	73
The absorption and emission of radiation	72
The Pauli exclusion principle	72
Infrared spectroscopy	71
Hydrogenic atoms	67
Vibration–rotation spectra	66
Many-electron atoms	65
The Born interpretation	65
Orthogonality	64
Angular momentum	63
Selection rules and transition moments	63
Molecular orbital theory of heteronuclear diatomic molecules	63
Rotational energy levels of molecules	62
Fluorescence and phosphorescence	60
The origins of quantum mechanics	59
Molecular orbital theory of polyatomic molecules	57
Translational motion in one dimension	56
The spectra of hydrogenic atoms	52
Experimental techniques in molecular spectroscopy	51
Tunnelling	50
The variation principle	50
The building-up principle	50
Superpositions	50
Rotation in three dimensions	49
Anharmonicity	48

Periodic trends of atomic properties	47
The spectra of many-electron atoms	47
Electronic spectra of Polyatomic molecules	46
Normal modes	46
Rotation in two dimensions	45
9B Molecular orbital theory of heteronuclear diatomic molecules	45
Electronic spectra of Diatomic molecules	44
Symmetry analysis of vibrational spectroscopy	40
Semi-empirical and ab initio methods	39
Symmetry operations and symmetry elements	39
Hermitian operators	39
The Beer-Lambert law	38
Valence-bond theory	38
Density functional theory	37
Vibrational Raman spectra	37
Self-consistent-field orbitals	36
Photoelectron spectroscopy	36
The Hückel approximation	35
Applications of group theory	35
The symmetry classification of molecules	34
Lasers	32
Microwave spectroscopy	29
Group theory	29
Dissociation and predissociation	24
Lifetime broadening	24
Nuclear magnetic resonance	24
Rotational Raman spectroscopy	21
Nuclear statistics and rotational states	19
Electron paramagnetic resonance	16
Doppler broadening	10
Pulse techniques in NMR	7
Solid-state NMR	6
